# Isoflurane's Effect on Protein Conformation as a Proposed Mechanism for Preconditioning

**DOI:** 10.1155/2011/739712

**Published:** 2011-09-12

**Authors:** Michelle R. Baker, Sean K. Benton, Christopher S. Theisen, Chad A. McClintick, Eugene E. Fibuch, Norbert W. Seidler

**Affiliations:** ^1^Department of Anesthesiology, University of Missouri-Kansas City School of Medicine, 4401 Wornall Road, Kansas City, MO 64108, USA; ^2^Department of Biochemistry, Kansas City University of Medicine and Biosciences, 1750 Independence Avenue, Kansas City, MO 64106, USA

## Abstract

Persistent alteration of protein conformation due to interaction with isoflurane may be a novel molecular aspect of preconditioning. We preincubated human serum albumin with isoflurane, dialyzed to release agent, and assessed protein conformation. Susceptibility to chemical modification by methylglyoxal and nitrophenylacetate was also examined. Isoflurane had a persistent effect on protein conformation. An increase in the susceptibility of surface residues to chemical modification attended this change in conformation. Modification of isoflurane-treated HSA included intra- and intersubunit cross-linking that may be a consequence of anesthetic-induced changes in multimeric subpopulations. This irreversible effect of isoflurane may represent a mechanism for preconditioning.

## 1. Introduction

The prevailing view of inhaled anesthetics is that they exhibit their desired effects on consciousness and response to pain by binding to target neuronal proteins [1]. The specific conformational changes that occur on binding of inhaled anesthetics remain an area of substantial research interest. Crystallographic and biochemical data reveal that binding sites exhibit considerable heterogeneity [2, 3]. Nevertheless, certain commonalities may exist [4]. The binding of inhaled anesthetics appear to affect interfacial regions in proteins that may be at sites interfacing two domains or subunits [1, 5]. These sites are considered to be less hydrated than substrate binding sites [6, 7]. Occupancy of these cavities by inhaled anesthetics may play a role in limiting conformational exploration of proteins. We think that upon release of anesthetic agents from these sites, a persistent alteration in conformation may occur that contributes to preconditioning.

The susceptibility of proteins to chemical modification by carbohydrate and lipid fragmentation products increases following exposure to inhaled anesthetics [8, 9]. The current study further explores the consequence of isoflurane binding using serum albumin as a surrogate protein in order to elucidate the effects of inhaled anesthetics on protein conformation. Human serum albumin (HSA) has several well-characterized anesthetic-binding sites [2, 3]. We think that the anesthetic-induced increase in susceptibility to chemical modification, such as glycation, may ultimately lead to cell signals associated with preconditioning. Interestingly, inhaled anesthetics upregulate heat shock proteins [10], which are involved in repairing protein misfolds. The unfolded protein response is thought to play a part in conferring cellular preconditioning [11]. We previously observed that protein unfolding increases the susceptibility to glycation [12], suggesting that susceptibility to chemical modification is dependent upon the conformational integrity of the protein. 

In the present study we tested whether glycation and acetylation of HSA is affected by isoflurane. Methylglyoxal (MG), a reactive di-carbonyl, is a glycating agent elevated in diabetics [13], and *p*-nitrophenylacetate (NPA) is a potent synthetic acetylating reagent that reacts with lysine residues that are common targets of monocarbonyl glycating agents. Albumin exhibits an esterase-like activity [14, 15], which recently has been redefined as chemical modification via acetylation of multiple sites by NPA that occurs in a biphasic manner with specific residues being sequentially modified [16]. We also looked at the recovery of protein conformation following extensive dilution of isoflurane-treated HSA. In addition to describing the appearance of a persistent isoflurane-directed misfold, we discuss the role of isoflurane on HSA oligomerization and MG-induced crosslinking. Our results suggest a novel mechanism of preconditioning, which is a curious attribute of isoflurane [17, 18].

## 2. Materials and Methods

### 2.1. Chemicals and Reagents

Unless otherwise stated, HSA (Sigma-Aldrich; A-8763, fraction V) solutions (150 *μ*M) were prepared in a 20 mM sodium phosphate (pH 7.4) buffer. Stock bottles of MG (Sigma-Aldrich; M-0252) and isoflurane (Hospira Laboratories, Lake Forest, IL; lot #35-526-DK) were used within three months after opening; MG (40% in water) was kept at 4°C and isoflurane was purged with nitrogen gas following use. *p*-Nitrophenylacetate (NPA) was purchased from Sigma (N8130) and freshly prepared (37.5% acetone) for each experiment. 8-Anilino-1-naphthalene sulphonic acid (ANS) was from Sigma. Water was deionized using Millipore's Milli-Q system to 18.2 MΩ/cm. All other chemicals were of reagent grade or better.

### 2.2. Exposure of HSA to Isoflurane

Unless otherwise indicated, HSA (950 *μ*L of 150 *μ*M) was treated with isoflurane (500 *μ*L) at room temperature by gently mixing in air-tight vials (Supelco) for 30 min prior to brief centrifugation and removal of upper aqueous phase containing HSA. Isoflurane is immiscible in water with a preference for the gas phase. The aqueous isoflurane concentrations were therefore determined using the ideal gas law and the partition coefficient for isoflurane in water/gas as described in [19]. Briefly, the partial pressure of isoflurane (ppI) in the air compartment of the vial (*gV*_*i*_) was calculated using the following equation:

(1) $$\text{ppI}=gV_{i}\times 760\;\text{mmHg}.$$

The ideal gas law, *PV* = *nRT*, where *P* is ppI, *V* is volume of the headspace (hs*V*), *T* is temperature in kelvins, and *R* is the gas constant (62.363 L · mmHg · °K^−1^ · mol^−1^), was used to determine the number of moles of isoflurane in the gas compartment (*n*(*g*)):

(2) $$n\left(g\right)=\frac{\left(\left(\text{ppI}\right)\cdot \text{hs}V\right)}{RT}.$$

The water/gas partition coefficient (*λw*) for isoflurane was used to calculate the amount of moles of isoflurane in the water compartment (*n*(*w*)):

(3) $$\lambda w=\left[\frac{\left(n\left(w\right)\right)}{\left(n\left(g\right)\right)}\right].$$

The concentration of isoflurane in the water compartment (*C*(*w*)) was obtained by dividing the number of moles of isoflurane (*n*(*w*)) by the volume in liters of the water compartment (*lV*_*w*_) in the vial and adjusted to mM as indicated in the following equation:

(4) $$C\left(w\right)=\left(\frac{n\left(w\right)}{lV_{w}}\right)\cdot 10^{3}.$$

Under these treatment conditions (950 *μ*L of HSA at 150 *μ*M and 500 *μ*L of isoflurane) with a defined headspace, the calculated concentration of aqueous isoflurane was 0.3 mM.

### 2.3. ANS Binding Assay

Following treatment of HSA (900 *μ*L of 75 *μ*M HSA with 300 *μ*L isoflurane for 1.75 hr at room temperature), which calculated to an isoflurane concentration of 0.4 mM, samples (600 *μ*L) were dialyzed against 500 mL sodium phosphate buffer (20 mM, pH 7.4) overnight with one change in dialysis medium. Dialysis was performed to remove bound isoflurane. Samples were then tested for ANS binding using fluorescence spectroscopy. HSA (1.5 *μ*M) was incubated with ANS (20 *μ*M) prior to obtaining fluorescence emission spectra from 400 to 600 nm with excitation at 360 nm, slit 10 nm. Background ANS emission was subtracted from resulting spectra. In determining the areas under the curves, intensities were integrated over the full range (400–600 nm), and controls were compared to isoflurane-treated samples using Student's *t*-test.

### 2.4. Dilution of Isoflurane-Treated HSA

The effects of dilution of HSA on the release of isoflurane following treatment were examined using intrinsic protein fluorescence. HSA contains a single tryptophan residue (W214) that allows for assessment of protein conformation. Isoflurane affects fluorescence spectra [5, 9], allowing for an indirect measurement of bound isoflurane. Spectra of samples of HSA (150 *μ*M) that were treated with isoflurane (0.4 mM for 35 min at room temperature) were obtained before and after extensive dilution. Emission spectra of samples were determined at 310–430 nm (slit 2.5 nm) with excitation at 293 nm (slit 2.5 nm). Values for the center of spectral mass were calculated from the emission spectra using the equation:

(5) $$\text{CSM}=\frac{\sum \left(\nu Fi\right)di}{Fidi},\;i=33.3\;{\text{cm}}^{-1},$$

where (*ν*) is wavenumber and (*Fi*) is emission intensity. They indicate spectral position and reflect the conformational events that change hydration at the W214 [20], suggesting conformational and oligomeric changes [5].

### 2.5. HSA Acetylation

Spectrophotometric assays were used to track the release of nitrophenol, which absorbs at 401 nm, following the acetylation of HSA by NPA. Solvent-directed hydrolysis of NPA was determined in the absence of HSA and subtracted from calculated acetylation rates. There were two phases of HSA acetylation: an early burst phase following by a late slow steady-state phase. The analysis of the early burst phase gave kinetics of acetylation rates of the residue Y411 [16]. This rate is equivalent to that was previously thought to be associated with burst-phase esterase activity [14]. The rate constant for Y411 acetylation was determined using the following second-order rate constant equation generating a straight line with the slope equal to *k*_2_:

(6) $$\begin{array}{cc} \log\;\;\left[\frac{\left(\text{NPA}i-\text{NP}\right)}{\left(\text{HSA}i-\text{HSA-A}\right)}\right] & =\left(\frac{\text{NPA}i-\text{HSA}i}{2.3}\right)k_{2}t\\ & \;-\log\;\;\left(\frac{\text{HSA}i}{\text{NPA}i}\right), \end{array}$$

where NPA*i* is the initial concentration of NPA, NP is the released nitrophenol measured over time, HSA*i* is the initial concentration of HSA, HSA-A is the acetylated HSA, *k*_2_ is the second order rate constant (M^−1^ min^−1^), and *t* is time (min). Maximum Y411 acetylation was determined from the *y*-intercept of the curve extrapolation. The late slow steady-state release of NP was used to measure the rate of surface residue acetylation using pseudofirst-order kinetics:

(7) $$\frac{d\text{NP}}{dt}=k_{1}^{\prime }\left[\text{NPA}\right]$$

to determine *k*_1_′ (1/min) after subtracting solvent-directed NPA hydrolysis.

### 2.6. Modification by MG

Control and isoflurane-treated samples of HSA (15 *μ*M) were then incubated with MG (0, 150, and 300 *μ*M MG for 18 hr at room temperature) under constant mixing in a 20 mM sodium phosphate, pH 7.4 buffer containing 1% methanol as an antimicrobial agent. Samples were then dialyzed (Spectrum Laboratories Rancho Dominguez, CA) using a Spectra/Por CE, 10 kDa MWCO (1 : 1000) against buffer for 3.5 hr prior to analysis.

### 2.7. AGE Fluorescence

HSA samples were treated and incubated as described above, and MG-induced fluorescence due to protein bound advanced glycation endproducts (AGEs) [9, 21] was measured from emission spectra that were integrated over 380–500 nm (slit: 2.5 nm) with excitation at 330 nm (slit: 5 nm). Duplicate scans were averaged using a speed of 400 nm/min with the filter cutoff set to open. Data were given in relative fluorescence units (rfu).

### 2.8. SDS PAGE

Control and isoflurane-treated HSA samples that were incubated with and without MG were dialyzed as described above and were electrophoresed under denaturing conditions using precast (Bio-Rad; Hercules, CA) Tris-HCl gels (7.5% polyacrylamide). Gels were run at constant voltage of 200 V, set at 60 mA for 45 min, fixed in 10% TCA, stained with Coomassie Blue R-250, and destained in 7% acetic acid, 10% methanol. The gels were then scanned and processed using SigmaGel, setting threshold levels at fixed points from baseline and integrating intensities.

### 2.9. Statistical Analysis

Student's *t*-tests (two-tailed, unless otherwise indicated) were conducted using SigmaPlot, 9.0 or Excel to determine significant differences using a 95% confidence limit. Linear regression analysis was done using SigmaPlot, and Pearson *r* coefficients were assessed using standard reference tables applying a two-tailed test. One-way ANOVA and Bonferroni post hoc tests were performed using SigmaStat to compare the blank data to the sample means.

## 3. Results

### 3.1. Isoflurane-Induced Persistent Misfold of HSA

**Figure 1:**
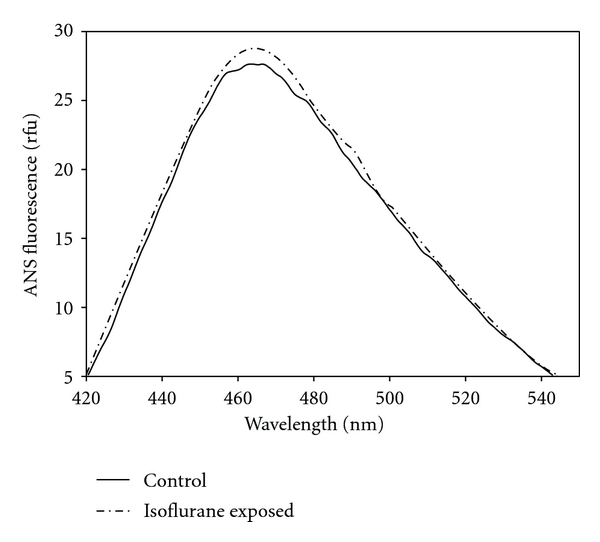
ANS binding to HSA after isoflurane exposure. HSA (75 *μ*M) was exposed to isoflurane (0.4 mM for 1.75 hr at room temperature), dialyzed, and examined for ANS binding versus untreated control. Data are representative samples.

After exposure of HSA (0.4 mM of isoflurane for 1.75 hr at room temperature under gentle agitation) and then dialysis to remove bound isoflurane, we observed a persistent change in protein conformation (Figure 1) as represented by increased ANS binding. This suggests that upon isoflurane release from their HSA binding sites, hydrophobic regions remained accessible.

**Figure 2:**
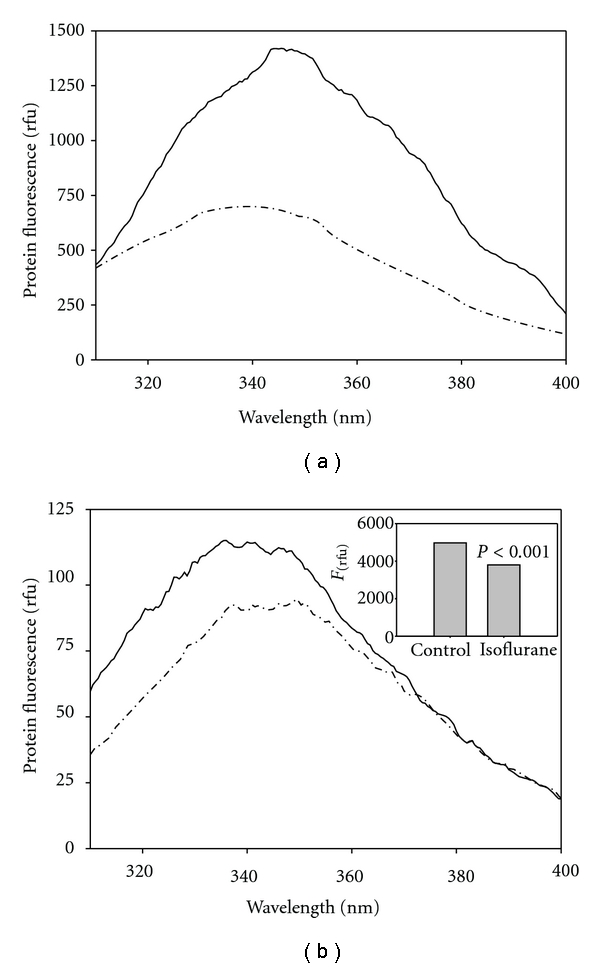
Comparison of fluorescence emission spectra after substantial dilution of HSA. Spectra before (a) and after (b) dilution: control (solid line) and isoflurane-treated (dash-dotted). Data obtained from representative samples. Spectra were integrated over 310 to 400 nm to obtain areas under the curve and compared (inset). Undiluted samples were measured at 1% attenuation of emission signal.

In a separate experiment, we diluted isoflurane-exposed HSA by greater than 30-fold (calculated final concentration of isoflurane: <10 nM) prior to measuring intrinsic fluorescence. Undiluted HSA (150 *μ*M), which was pretreated with isoflurane (0.4 mM for 35 min at room temperature), exhibited a large spectral difference relative to control (Figure 2(a)). Upon dilution, we observed that total fluorescence intensity fell below 100% of control (Figure 2(b)) suggesting that complete recovery of native conformation did not occur. It also appeared that the spectra of the diluted, treated samples were red-shifted relative to control (CSM values: 28927.6, control versus 28664.9 cm^−1^, isoflurane), suggesting improper refolding of HSA upon isoflurane release. It is important to note that upon isoflurane binding to HSA, there is a blue-shift in the spectra (Figure 2(a)), indicating the release of isoflurane caused a structural change in the area associated with the anesthetic binding site as the protein refolds.

### 3.2. Acetylation of HSA Residue Y411

**Figure 3:**
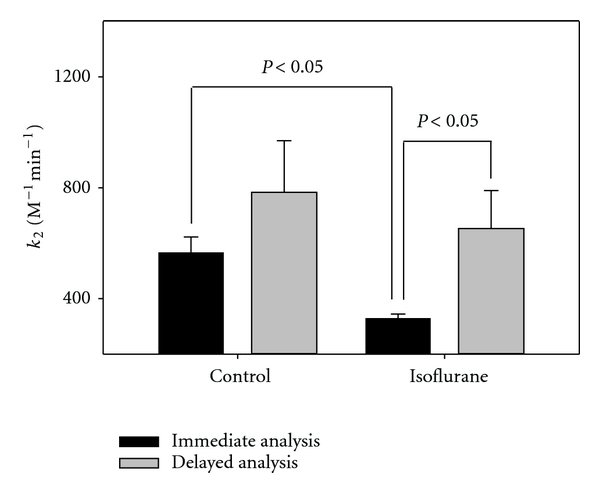
Acetylation of residue Y411. NPA-induced acetylation was measured following dilution of isoflurane-treated HSA. Samples were either analyzed immediately after dilution (black bars) or there was a timed delay of at least 20 min following dilution (gray bars). Data are given as mean values ± SEM obtained from multiple measurements.

NPA acetylates residue Y411 of HSA as measured by burst phase analysis described in Section 2. We tested the effects of isoflurane on Y411 acetylation. HSA (150 *μ*M) was first treated with isoflurane (0.5 mM for 70 min at room temperature), diluted 30-fold, and analyzed immediately (<20 min) for NPA-induced acetylation. We observed that isoflurane inhibited acetylation of Y411 (Figure 3, black bars). The control rate constant (*k*_2_) from immediate analysis was 566 ± 56 versus isoflurane treated (330 ± 14 M^−1^ min^−1^, *P* < 0.05). Interestingly, when samples were kept longer than 20 min after dilution, the inhibition of Y411 acetylation was no longer observed, suggesting that isoflurane's effect was reversible. The observed difference between immediate (330 ± 14) and delayed (653 ± 137, *P* < 0.05) analysis of the isoflurane-treated samples supports this interpretation. It was previously shown that isoflurane binds at low millimolar affinity to the hydrophobic cavity that contains the Y411 residue [3]. Our results are consistent with the time-dependent release of isoflurane from the Y411 site after dilution. Additionally, no difference was observed between control and treated samples in maximum Y411 acetylation (data not shown) as determined by curve extrapolation to the *y*-intercept as described in the Section 2. This also supports the notion that the binding of isoflurane to this particular region of the protein is labile.

### 3.3. Modification of Surface Residues on HSA

**Figure 4:**
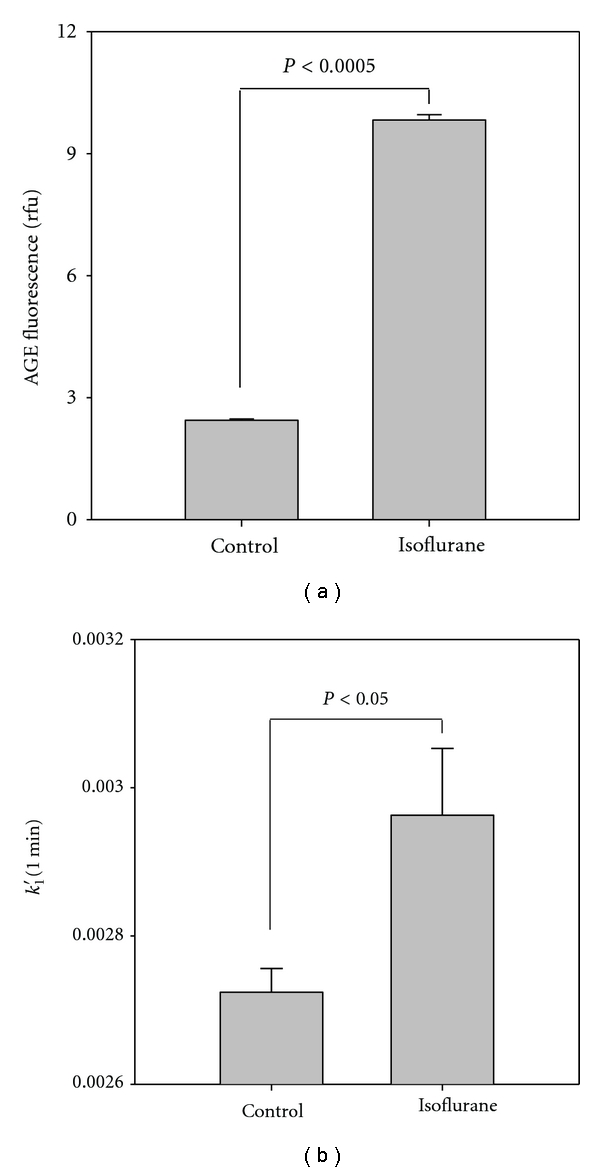
Isoflurane increases surface residue modification. HSA was treated with isoflurane prior to dilution and analysis of MG-induced formation of AGEs (a) and NPA-induced acetylation (b). The measurements were delayed and indicate an isoflurane-induced effect that was not labile. Data are presented as mean values ± SD from 2–4 experiments.

We were also interested in examining the effect of isoflurane on the modification of other HSA residues, such as surface residues that may be modified by NPA as well as MG. We observed that isoflurane increased the susceptibility of HSA to chemical modification by MG (Figure 4(a)). HSA (150 *μ*M) was treated with isoflurane (0.3 mM for 15 min at room temperature) prior to 1 : 10 dilution and exposure to MG (300 *μ*M for 18.5 hr at room temperature). MG-induced fluorescence (ex: 330 nm; em: 380–500 nm), which is due to the formation of advanced glycation endproducts (AGEs) particularly argpyrimidine, increased by 4-fold after isoflurane exposure (2.4 ± 0.03 to 9.8 ± 0.13 rfu, *P* < 0.0005). We previously observed that isoflurane increased the susceptibility of glyceraldehyde 3-phosphate dehydrogenase (GAPDH) to MG-induced modification [9]. Acrolein-induced modification of HSA was also promoted by trifluoroethanol that is used to mimic the effects of inhaled anesthetics on protein structure [8]. Our observations with MG represent modifications of residues that are rather accessible, suggesting that they are exposed to solvent and likely on the surface of the protein. The levels of MG used in these experiments are physiological and consistent with those previously shown to minimally modify HSA with MG [22]. We also observed that rate of NPA-induced acetylation of surface residues was also enhanced after treatment of isoflurane (Figure 4(b)). HSA (150 *μ*M) was treated with isoflurane (0.6 mM for 60 min at room temperature) prior to 1 : 37.5 dilution and measurement of acetylation rates as described in Section 2. The pseudofirst-order rate constant for acetylation (*k*_1_′) increased by 9% with isoflurane. The observations of MG- and NPA-induced HSA modification (Figures 4(a) and 4(b)) represent a persistent effect of isoflurane that is sustained after dilution and delayed analysis. This is consistent with the small irreversible change in native protein conformation (Figures 1 and 2).

### 3.4. Changes in Electrophoretic Mobility of MG-Modified HSA Treated with Isoflurane

**Figure 5:**
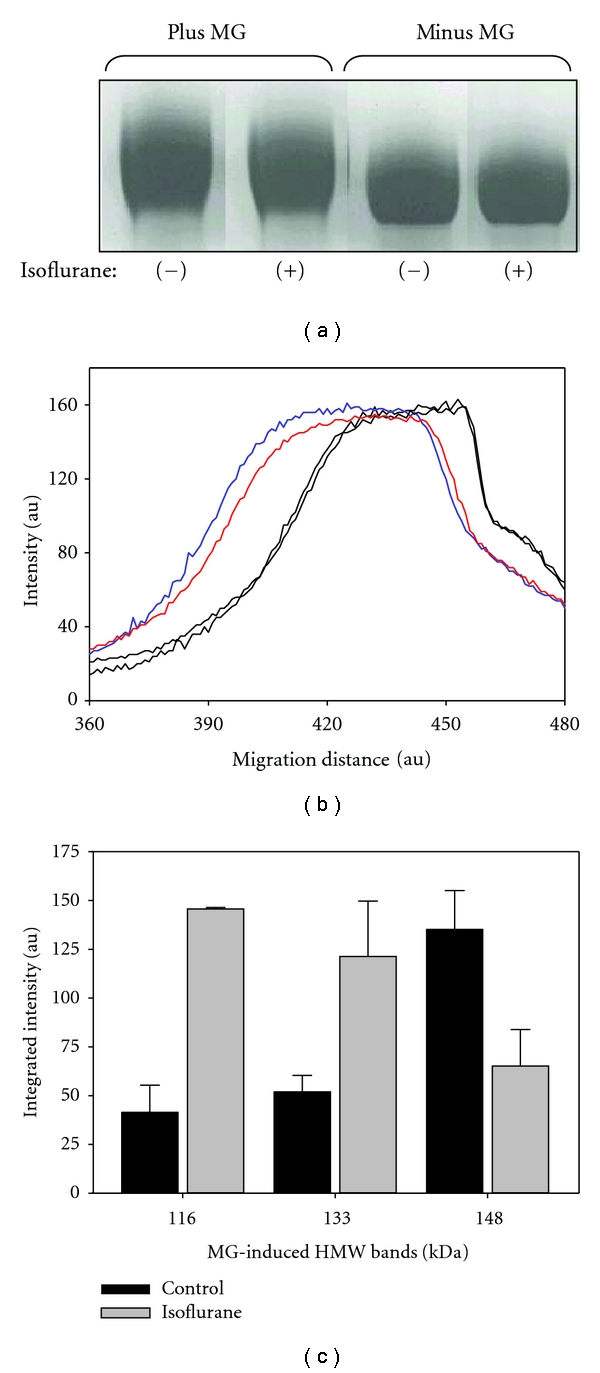
Electrophoretic mobility of MG-modified HSA treated with isoflurane. HSA was pretreated with isoflurane (0.3 mM) prior to dilution and exposure to MG (3 mM for 7 days at room temperature). Following SDS-PAGE, analysis of Coomassie-stained gels involved visual inspection (a) and densitometry (b) of the major 66 kDa band. Additionally, the minor MG-induced HMW bands (c) were analyzed by integration of densitometric scans using SigmaGel. The densitometric analysis shown in (b) presents the migration changes of the major bands in (a). The migration distance in (c) represents arbitrary units of measurement that originate from the top of the gel. The color tracings are the plus MG samples that were treated (red) and untreated (blue) with isoflurane. The black tracings are the respective minus MG samples.

When HSA was incubated with MG (0.5–3.0 mM for 7 days at room temperature), we observed that the migration of the 66 kDa HSA bands on SDS-PAGE gels (7.5%) was slightly altered (Figure 5). MG caused a similar shift in the leading edge of the HSA band in both isoflurane-treated (0.3 mM) and untreated samples. However, the lagging edge differed. The MG-modified HSA band in the isoflurane samples appeared less diffuse than control MG-modified HSA. The visual observations seen in Figure 5(a) were substantiated upon densitometric analysis (Figure 5(b)). These results suggest that MG-induced intramolecular crosslinking may be enhanced by isoflurane, resulting in more compact denatured structures that have slightly altered electrophoretic mobility. Additionally, there are minor detectable amounts of high molecular weight (HMW) bands (apparent MW: 116, 133, and 148 kDa) found in control that may be attributed to *in vivo *modifications as previously mentioned [22] that may lead to subunit-subunit crosslinking. Further substantiation of *in vivo *modifications of HSA is found in the common fructosamine blood test, which measures serum levels of glycated albumin. We observed that MG increased the formation of all three minor HMW bands. The data shown in Figure 5(c) represents the subtraction of background intensity of minus MG controls. 

The increase above control suggests that these minor HMW bands are derived from crosslinking of the major 66 kDa subunit. Our current working model is that the 116 and 133 kDa bands represent cross-linked HSA dimer species and that the 148 kDa represents a cross-linked HSA trimer. This observation is consistent with the appearance of HSA dimers and trimers at these concentrations [23]. Isoflurane altered the distribution of the MG-induced HMW bands, promoting the formation of 116 and 133 kDa bands while decreasing the formation of the 148 kDa band (Figure 5(c)). Interestingly, we previously observed that sevoflurane and isoflurane promoted HSA dimerization while inhibiting oligomerization, likely through anesthetic-induced trimer destabilization [5].

## 4. Discussion

We report that isoflurane's effect on protein conformation is persistent with isoflurane's release occurring during dilution of HSA as evidenced by ANS binding (Figure 1) and W214 fluorescence after dilution (Figure 2). Isoflurane was previously shown to bind to this region, which is at the interdomain cleft of HSA [3] a protein that is composed of three homologous domains (I–III) each with two like subdomains (A, B). W214 is found at domain IIA, which is found adjacent to IIB at the vertex of the bilobed protein—a region thought to be involved in oligomeric interactions [5]. Consistent with the recovery of signal at the W214, isoflurane's inhibitory effect on acetylation of Y411 was labile and was lost upon dilution and prolonged incubation (Figure 3). This suggests that isoflurane exhibits a low-affinity binding to the Y411 region, which is in domain IIIA and known to contain a large internal hydrophobic cavity [3]. Isoflurane may block Y411 acetylation that is lost upon release of isoflurane from this internal pocket, which is likely not involved in subunit-subunit interactions.

**Figure 6:**
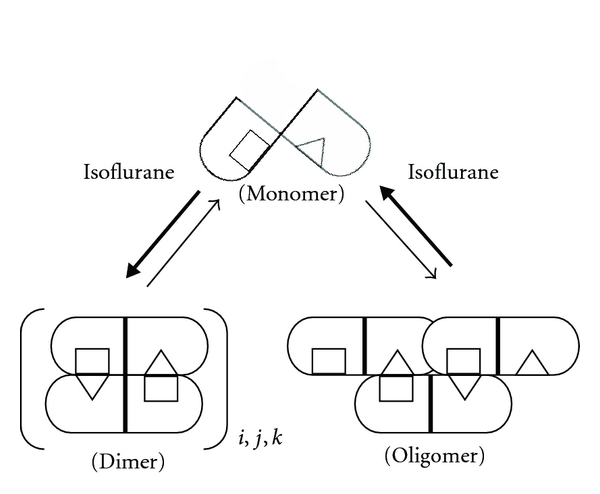
Target HSA species that are modified by MG. Our model suggests that the HSA dimer represents an isologous association of twofold symmetry with complementary binding sites indicated by squares and triangles. The nucleus for further higher-order HSA oligomerization may be a trimer [24] with a proposed screw symmetry, forming a heterologous association. While isoflurane promotes dimerization [5], it may also affect the distribution of HSA dimer subpopulations (indicated by italicized subscripts: *i, j,* and* k*). Intra- and intermolecular crosslinking by MG (as well as surface residue acetylation) may be influenced by subunit interaction and lead to cellular responses conferring preconditioning.

Upon extensive dilution of HSA after isoflurane treatment, we observed a residual persistent effect on the fluorescent properties of HSA (Figure 2(b)), suggesting an irrecoverable change in conformation that is recognizable at the W214. The total fluorescence intensity of the isoflurane-treated samples remained less than control, and the spectra changed from a left-shifted to a right-shifted position compared to control after extensive dilution. The spectral overshoot may be due structural changes that result in increased hydration surrounding the W214 residue in domain IIA as indicated by the postdilution right shift (Figure 2(b)). This may represent a persistent misfold that may translate to preconditioning signals, such as induction of stress-response pathways. The right-shift may also reflect the favored dimer configuration of isoflurane-treated HSA as previously suggested [5]. HSA is present as dimers and higher-order structures beginning with trimers and progressing incrementally to tetramers, pentamers, and larger oligomers [23]. We previously proposed that the HSA dimer occurs through reciprocal interchange of subdomains IIA (helix 2 and 3, residues 208–247) and IIB (helix 2 and 3, residues 323–362) from two monomers [24], forming an isologous association. A reconfiguration would be required to construct multimers via alternating interchanges resulting in a concatemeric linkage via the internal sequences at subdomains IIA/IIB in a heterologous association. A crosslinking study [25] using homobifunctional linkers found a crosslink between K162 of one subunit and K313 of an adjacent subunit, consistent with our proposed model (Figure 6).

Considering that isoflurane promotes the formation of HSA dimers and destabilization compact oligomers [24], one would expect an increase in the solvent-exposed surface residues in the isoflurane-treated samples relative to control. Consistent with this model, we observed that surface arginine and lysine residues on isoflurane-treated HSA were more susceptible to modification by MG and NPA than those of control (Figure 4). There are over 80 accessible residues, including all 59 lysine residues that are potentially modified by NPA [16]. There are 24 arginine residues of which five are readily modified (R114, R186, R218, R410, and R428) [22]. MG produces several types of AGEs [21]. We tracked the production of MG-derived AGEs that fluoresce, a characteristic typically attributed to argpyrimidine [8, 26]. These fluorophores correlate with the formation of other AGEs, such as hydroimidazolone [27], and they represent advanced rearrangements of early unstable reaction products. Interestingly, host HSA from normal and diabetic individuals contains some glycation products [22, 28]. The surface residues on HSA described above may be associated with regions involved in subunit-subunit interactions.

The effect of isoflurane on these surface regions is consistent with our previous model of the binding of inhaled anesthetics to interfacial sites of multimeric proteins thus altering their oligomeric states [5, 9]. The HMW bands of HSA that migrated at 116, 133, and 148 kDa may represent MG-induced lysl-lysl or lysl-arginyl cross-linked species of HSA. Interestingly, the distribution pattern of these three species of MG-modified HMW bands shifted to the 116 and 133 kDa species in isoflurane-treated HSA (Figure 5(c)). This suggests that the 116 and 133 kDa species may represent cross-linked dimers, consistent with the observation that isoflurane promotes HSA dimerization [5]. The 148 kDa species is dominant in control over isoflurane-treated samples, consistent with this form representing a putative cross-linked trimer, which may have migrated further than the expected 198 kDa due to the nature of its oligomeric configuration. We previously proposed that the oligomer, which stems from a trimeric nucleus, has a more compact configuration with screw symmetry [24]. Crosslinking along two adjacent helical spans, for example, may constrain the cross-linked product from become completely linear unfolded chains under denaturing conditions, thus yielding altered electrophoretic mobility.

Arginine residues (and secondarily lysine residues) are the preferred nucleophilic centers for MG modification [21, 22]. Interestingly, four of the eight halothane binding sites contain arginine residues (site 1: R209; site 2: R209; site 5: R257; site 6: R218; R222) [2], suggesting that isoflurane may alter the microenvironment around these sites as well as promote more distant conformational changes that affected the susceptibility to MG modification. The HSA monomer bands in SDS-PAGE that represent MG modification appeared less diffuse in the isoflurane-treated samples (Figures 5(a) and 5(b)), suggesting that tertiary changes in protein structure may impact the profile of residues that are modified. The reactivity of MG to the arginine residues found near the anesthetic binding sites may be affected upon isoflurane binding, thus changing the heterogeneity of the MG-modified HSA monomer bands.

Preconditioning by isoflurane is known to be neuroprotective [17, 18], suggesting that isoflurane may contribute to a nonlethal “injury” that provides some benefit to the host cell. We observed that HSA, which was treated with isoflurane and subsequently dialyzed or diluted, resulted in a persistent effect on protein conformation (Figures 1 and 2(b)). There may be an isoflurane-directed protein misfold upon dilution that may generate a conformational stress to the cell initiating protective pathways for protein folding. Our laboratory is currently exploring this model.

In summary, we used HSA as the surrogate protein to examine the molecular interactions associated with the binding of inhaled anesthetics and changes in susceptibility to chemical modification. Isoflurane's effect on conformation was largely reversible with a small residual alteration that persisted after extensive dilution. Concurrent with these effects on conformation, we observed labile changes in susceptibility to chemical modification as well as more persistent changes in susceptibility involving enhanced reactivity by acetylating agent, NPA, and glycating agent, MG. These observations are consistent with previous findings that inhaled anesthetics increase susceptibility of proteins to chemical modification [8, 9]. We also observed an isoflurane-dependent effect that may be, through modulation of HSA subpopulations, an observation consistent with our previous model of inhaled anesthetics binding to interfacial sites of multimeric proteins [5, 9]. We think that the slight irreversible effect of isoflurane may represent a mechanism for preconditioning, suggesting that isoflurane may induce stress-response pathways by altering protein conformation and susceptibility to chemical modification.
